# Application of the Kvaal Method in Age Estimation of the Serbian Population Based on Dental Radiographs

**DOI:** 10.3390/diagnostics12040911

**Published:** 2022-04-06

**Authors:** Dejan Zdravkovic, Milica Jovanovic, Milos Papic, Vladimir Ristic, Andjela Milojevic Samanovic, Aleksandar Kocovic, Miroslav Sovrlic, Miona Vuletic, Aleksandra Misic, Rasa Mladenovic, Marko Milosavljevic, Jelena Todic

**Affiliations:** 1 Department of Dentistry, Faculty of Medical Sciences, University of Kragujevac, 34000 Kragujevac, Serbia; zdravkovicdejan91@yahoo.com (D.Z.); micamonro@gmail.com (M.J.); milos_papic@live.com (M.P.); vristic7@gmail.com (V.R.); andjela-kg@hotmail.com (A.M.S.); miona91kg@gmail.com (M.V.); kaja_ok@hotmail.com (A.M.); 2 Department of Pharmacy, Faculty of Medical Sciences, University of Kragujevac, 34000 Kragujevac, Serbia; salekkg91@gmail.com (A.K.); sofke-ph@hotmail.com (M.S.); 3 Clinic of Prosthodontics, Faculty of Medical Sciences, University of Pristina, 38220 Kosovska Mitrovica, Serbia; todic.j@gmail.com

**Keywords:** forensic dentistry, Kvaal method, age estimation, orthopantomography, dental radiographs

## Abstract

This study aimed to evaluate the applicability of the Kvaal method in determining the age of a Serbian population. In this retrospective descriptive study, 170 dental patients (age range 16–77 years) were included. The measurements of six types of teeth were performed on digital orthopantomography radiographs according to the Kvaal method. Statistical inter-observer and intra-observer reliabilities were calculated to evaluate the reproducibility of the measurements, as well as to examine the correlation between chronological age and measured values. The evaluation revealed a substantial difference (over 12 years) between chronological and estimated age. The P ratio had the strongest correlation with chronological age for the maxillary central (r = ±0.293) and lateral incisors (r = ±0.240), whereas the pulp/root width ratio at level A had the strongest correlation for the mandibular first premolars (r = ±0.270). The lowest SD values, for measurements on teeth 15/25 (SD = 125,787), had the most accurate correlation with age. Thus, we can conclude that the original Kvaal method is not applicable in the age estimation of the Serbian population. For future research, we suggest using cone beam computer tomography as a precise technology to evaluate the applicability of Kvaal and other methods for age estimation.

## 1. Introduction

Assessment of chronological age is one of the most important factors in determining the identity of living, dead, and those who died violently. The deceased can be identified based on bone structures and their growth and development [1]. For these purposes, the development of teeth can be useful. The growth and development of teeth in age determination is an applicable method by the age of twenty-five [2]. Until then, the growth and development of all teeth of the upper and lower jaw are expected to be completed. After the age of twenty-five, teeth and their anatomy go through minimal changes over the years, so certain specificities that could be associated with the chronological age of unidentified people are lost [3,4]. One of the methods used in these situations is the measurement of the amount of secondary dentin deposited inside the pulp chamber. This is a very invasive method, and it is not applicable to living subjects, since tooth extraction is most often required for its realization [5]. On the other hand, measurement of the amount of secondary dentin based on orthopantomographic (OPG) radiographs is possible, but the results have been shown to be less accurate [6]. Third molar mineralization is another method for age estimation in humans, though with certain limitations [7].

The fact that tooth enamel is the hardest tissue in the body and takes the longest to decompose can be useful for identifying people posthumously [8]. In these situations, their identity and approximate age are most easily established by teeth [5].

Many authors have tried to develop an adequate regression formula by measuring different teeth or groups of teeth in order to achieve a better prediction of the subject’s age [9,10,11].

Kvaal et al. [9] established a non-invasive method where, using the analysis of retroalveolar and OPG radiographs, based on the measurement of individual teeth at multiple points, a regression analysis leads to the conclusion of the approximate age. One of the advantages of this method is that it can be performed on both the living and the dead [9].

Numerous studies have reported that the Kvaal method has a direct correlation with chronological age, but variations in age estimation are observed in the populations it is applied to [12,13,14].

Research conducted on a Brazilian population had different results depending on the age of the patients, with the most accurate predictions of the subjects’ ages for the groups 20–29 and 30–39 years of age, unlike other age groups [15].

Similar results were obtained in a study conducted on a Malaysian population, which found that greater accuracy of the Kvaal method was present in the younger population, but despite the more accurate results, they still did not meet the criteria for application in assessing the age of the given population [16].

In addition to OPG recordings, the Kvaal method can also be implemented through cone beam computed tomography (CBCT). Although CBCT technology is more accurate than measurements conducted on OPG scans, the results so far do not favor greater accuracy in determining the age of subjects [17,18].

New technologies also provide new possibilities in daily practice. In recent years, it has become an increasingly common practice to record each patient’s digital data individually in electronic records that store data such as digital 2D and 3D recordings, photos, and intraoral scans. All these data can make it much easier to identify missing people; therefore, it would be desirable to increase the frequency of digital dental medical files [19].

Due to the numerous war events that occurred in many countries of Southeastern Europe in the past thirty years, there are many missing and dead people whose identity is still not established [20]. Southeastern Europe is also one of the routes that many immigrants pass through on their way to Western and Central Europe, which leads to their temporary or permanent settlement in Western Balkan countries, including Serbia [21]. For these reasons, it is desirable to determine whether the Kvaal method is applicable to the Serbian population in order to establish whether there is a difference in the prediction of age compared to measurements obtained on the population of other ethnicities.

The aim of this study was to investigate the applicability of the Kvaal method in determining the age of subjects in the Serbian population.

## 2. Materials and Methods

The study was designed as a retrospective descriptive study which includes the analysis of patient OPG radiographs taken at the Department of Dentistry, Faculty of Medical Sciences, University of Kragujevac, between June 2017 and June 2019. All OPG radiographs were obtained following the ALARA (as low as reasonably possible) principle using Orthophos XG (Sirona Dental Systems, D-64625, Bensheim, Germany), set at 62 kV–7 mA, with an exposure time of 14.1 s. Patient gender and age were retrieved from the registry.

### 2.1. Sample Population

The sample consisted of digital OPGs of 170 Serbian dental patients (64 males and 106 females). Subjects included in the study were in the age range of 16–77, with a mean age of 31.02 ± 12.11 years. Unlike Kvaal’s method which encompassed a population 20–87 years old, our sample included people aged 16 to 77. The reason for the lower limit of 16 years is that by the age of 16, root growth has already been completed, so there are no obstacles to including 16-year-olds in the study. In addition, younger patients have a lower incidence of composite or amalgam restoration, root canal treatment, tooth extraction, or major tooth abrasion or attrition, so subjects are more likely to meet the criteria for inclusion in the study. The upper limit of 77 years has been lowered compared to Kvaal’s method, where it was 87 years, due to the increasing incidence of toothless patients in the older population in Serbia, so a very small sample of patients in the range of 77–87 would be expected to meet the study criteria. Age distribution in females was 17–73 years (mean 29.69), and 16–77 years (mean 33.22) for males. A test group was extracted from the original sample. The test group consisted of 41 patients (21 female and 20 male) and was used for testing the obtained models.

### 2.2. Tooth Selection

**Figure 1:**
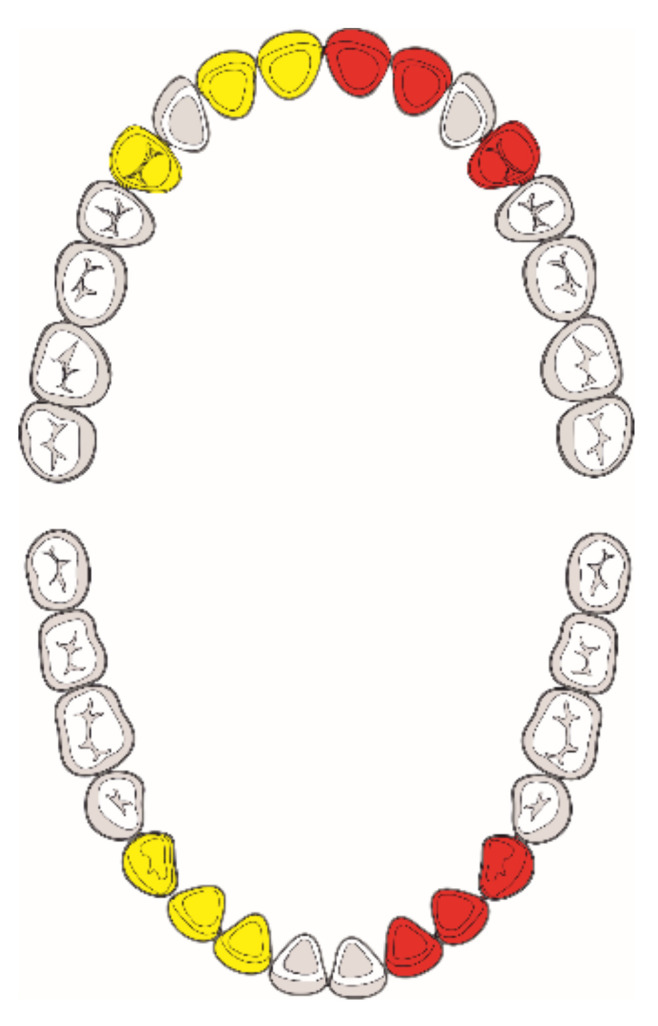
Teeth analyzed. Tooth selection was based on their presence on OPG radiographs. If present, teeth on the right jaw side (yellow) were analyzed; in their absence, their right counterparts (red) were selected for analysis.

According to the methods defined by Kvaal et al. [9], the teeth that showed the highest correlation with age were the maxillary central incisor, lateral incisor, and the second premolar; and a lateral incisor, canine, and the first premolar in the mandible. Given that there was not a major difference between the left and right teeth according to Kvaal et al. [9], we used these teeth for the analysis regardless of their position in the jaw (Figure 1).

### 2.3. Inclusion Criteria

The inclusion criteria were that the OPG radiographs presented the targeted teeth (11/21, 12/22, 15/25, 32/42, 33/43, 34/44) fully visible with the absence of caries, composite or amalgam restorations, root canal treatment, periapical lesions, tooth rotation, and major tooth abrasion or attrition. The targeted teeth had to be in a functional and healthy occlusion based on what we could tell from the characteristics spotted on the OPG radiographs. The absence of all the above factors for exclusion from the study and also the presence of antagonist teeth indicated that the tooth is in a functional and healthy occlusion. Additionally, teeth that had greater overlapping of the agonist teeth shadows were also excluded from the measurements [9].

After excluding the recordings of patients who did not fulfil the specified criteria, the remaining recordings were included in the study.

### 2.4. Measurements

**Figure 2:**
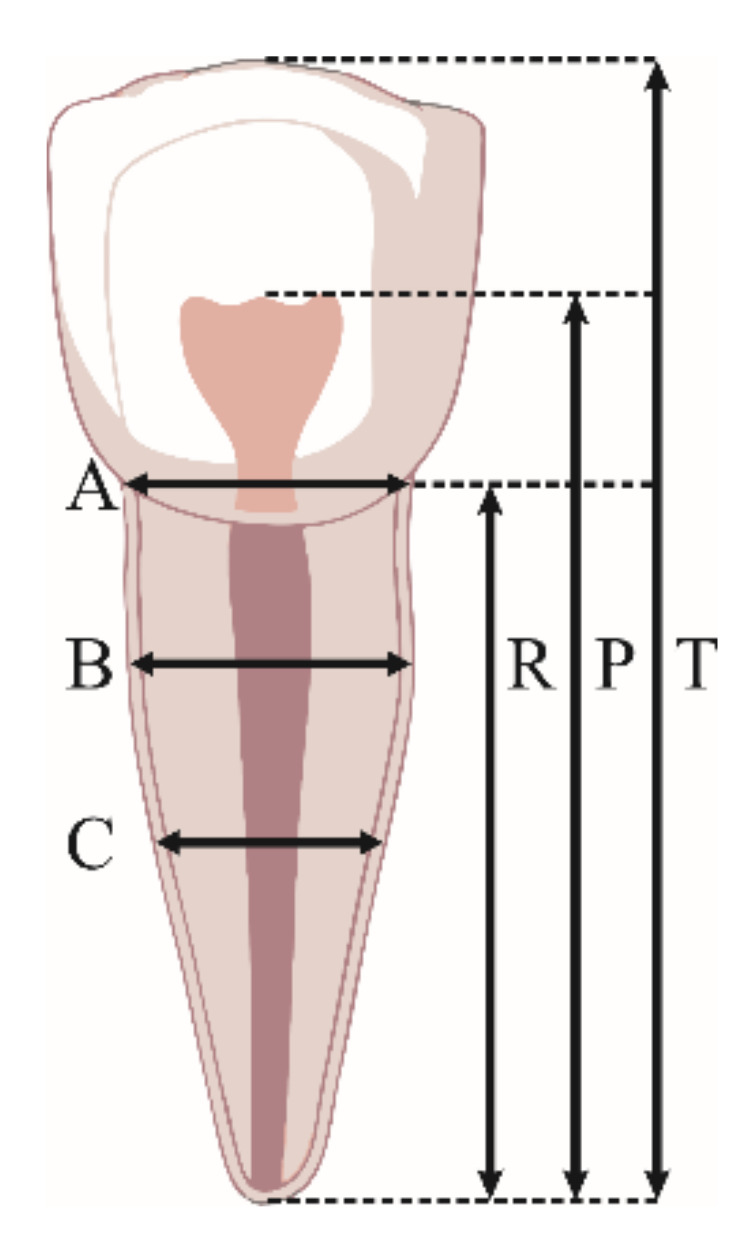
Tooth measurements performed in this study: T, maximum tooth length; P, maximum pulp length; R, maximum root length; A, root and pulp width at enamel–cementum junction; C, root and pulp width at mid-root level; B, root and pulp width halfway between levels A and C.

Measurements were performed on 170 OPG radiographs according to the criteria defined by Kvaal et al. [9] (Table 1). All OPGs were obtained in tiff format, and they were imported into the ImageJ program v1.53e (National Institutes of Health, Bethesda, MD, USA) where the final measurements were made. All radiographs were calibrated and magnified to 200% before the measurement started [22]. The following measurements were obtained on each of the selected teeth (Figure 2).

Based on these measurements, the following Kvaal dental ratios were calculated: the tooth/root length, the pulp/root length, and the pulp/tooth length, as well as the pulp/root width at the three levels [22].

Measurements were obtained by two investigators who are skilled in radiograph analysis using the software.

### 2.5. Statistical Analysis

Possible differences in tooth measurements by the same investigator were confirmed by repeating the measurements on five different OPG radiographs at five different intervals with a one-day pause between measurements, and adding three new OPG radiographs to each repeated measurement to exclude the possibility of self-suggestion when measuring.

The possible measurement error was then estimated by calculating the technical measurement error (TEM), the relative technical measurement error (rTEM), and the coefficient of confidence (R) following anthropometric standards [13].

Age and gender data were firstly analyzed using the methods of descriptive statistics (mean, range, standard deviation). For exploring the degree of linear association between age and Kvaal dental ratios, Person’s correlation coefficient was used. Multivariate regression analysis (backward method) was used to evaluate the relationship between Kvaal dental ratios and age. Ratios of individual teeth and the combination of ratios from different teeth were used to produce the most adequate statistical model. Multiple regression models were formulated using the standard method, whereby the independent variables (M and W-L values) entered the equations simultaneously to predict chronological age (dependent variable), as described previously [13]. In addition to tooth specific models, regression equations were formulated for the following tooth combinations:

(1) The three maxillary teeth with their individual M and W-L values (6 predictors), and averaged M and W-L values (2 predictors).
(2) The three mandibular teeth with their individual M and W-L values (6 predictors), and averaged M and W-L values (2 predictors).
(3) All six teeth with their individual M and W-L values (12 predictors), and averaged M and W-L values (2 predictors).
(4) Kvaal dental coefficients with the strongest correlation with age (10 predictors reduced to 5 predictors in the backward regression model).

In one regression analysis, dental ratios with the best correlation were used. The predictive accuracy of the models was quantified using the standard error of estimate (SEE) in the extracted cross-validation test sample [23]. All statistical analyses were performed using the Statistical Package for Social Sciences SPSS v. 20 (SPSS Inc., Chicago, IL, USA) for Windows.

## 3. Results

Both intra-observer (TEM = 0.34, rTEM < 5%, R = 0.78) and inter-observer error between investigators (TEM = 0.39, rTEM < 5%, R = 0.75) were acceptable, showing some difference in measurements between subjects, but their measurement accuracy was still comparable. Differences in the interpretation of digital OPGs happened due to differences in experience, mode, and training.

Pearson correlation coefficient was used for the assessment of linear correlation between age and Kvaal dental ratios. The correlation coefficients between age and Kvaal dental ratios are presented in Table 2 and Table 3.

The mean difference between chronological age and calculated age in years, standard deviation of mean values in years, standard error of the mean values in years, and *t*-test results are presented in Table 4. The lowest SD values, having the most accurate correlation with age, were for measurements on teeth 15/25 (SD = 12,5787).

The P ratio had the strongest correlation with chronological age for the maxillary central (r = ±0.293) and lateral incisors (r = ±0.240), whereas the pulp/root width ratio at level A had the strongest correlation for the mandibular first premolars (r = ±0.270). The correlations between chronological age and the M ratio were the highest for the maxillary central incisors (r = ±0.167), first mandibular premolars (r = ±0.160), and maxillary lateral incisors (r = ±0.133).

The individual tooth, multiple teeth, and the strongest correlation coefficient regression models are presented in Table 3 and Table 5, respectively.

Regression models that take maxillary teeth into account had higher prediction accuracy compared to models that consider mandibular teeth. It was observed that the regression models for the individual mandibular teeth consistently had lower prediction accuracy compared to the maxillary teeth (Table 6).

The most accurate model was the maxillary lateral incisors model (SEE ± 12.393 years), and the least accurate model was for the mandibular lateral incisors (SEE ± 12.700 years). The regression model with averaged M and W-L values (two predictors) for the maxillary teeth had a lower accuracy (SEE ± 12.534 years) than the model with individual M and W-L values (six predictors; SEE ± 12.269 years). Individual M and W-L values for all six teeth (12 predictors; SEE ± 12.120 years) had the most precise age prediction (Table 5). Using Kvaal dental ratios with the strongest correlation with age for model construction resulted in better parameters (higher R and R2 values and lower (absolute) SEE) (Table 6).

The accuracy of the derived regression equations (Table 5 and Table 6) was tested on a random sample of 41 OPGs (Table 7). Predictive accuracy was highest for the equation requiring three maxillary teeth (six predictors) (SEE ± 11.520 years). Although the regression equations in Table 6 have better values of the R, R2, and SEE parameters, they did not prove to be superior during control group testing (Table 7).

## 4. Discussion

Age estimation using the Kvaal method showed that there is a large disparity in the estimation of the subject’s age depending on the population it is applied to. This method has the potential to be applied to a wide population because it is non-invasive and easy to use. However, additional authors obtained results similar to ours, with a significantly larger error in age estimation than the results of Kvaal et al.’s study [9,12,22,24,25] (Table 8).

Although Kvaal et al. [9] used retroalveolar radiographs, the real application of this method on retroalveolar radiographs is difficult because of the number of images that are needed to perform measurements on all target teeth. For these reasons, measurements are mostly performed on OPG radiographs [12,23,24,26].

Using Kvaal’s original method, the age estimation error in this study was SEE = ±12.120 years when we used regression analysis for all six teeth at the same time (Table 5). Our results are similar to the results of Li et al. [12], who applied this method on a Chinese population (*n* = 360) (SEE = ±11.4 years). Furthermore, a significant age estimation error was obtained by Karkhanis et al. [13] for a population of Western Australia (*n* = 279); the smallest error in age prediction was made when regression analysis was performed for M and WL values for all six teeth (SEE = ±7963 years) [13].

Kanchan-Talreja et al. [25] compared two methods for intraoral radiography in the Indian population (*n* = 100); the parallel method, in which the film or sensor is held parallel to the radiation source by specific holder (paralleling technique), and another method where the film or sensor leans on the lingual/palatal surface of the tooth so that the radiation source is directed vertically on the detector (bisecting technique). In both techniques, the standard error was significantly high (SEE = ±8.6 years), although they used retroalveolar radiographs. The age estimation was similar to the results we obtained in our study if we look at the measurements of all six teeth (paralleling technique SEE = ±12.08 years, and bisecting technique SEE = ±11.9 years). Their study reported the most accurate results with the parallel method on the maxillary second premolar (SEE = ±11.87 years), and on the maxillary central incisor with the bisecting technique (SEE = ±11.17 years). There are no significant differences in measurements on OPG radiographs compared to intraoral retroalveolar radiographs according to these results [25].

When we compared the standard deviation (SD) obtained in this study with other published results, we concluded that they were similar (Table 8). Only two authors had more significant differences in SD values [26,27].

The study of Bosmans et al. [26], conducted on a Belgian population (*n* = 197), unlike most other authors, had more favorable results (SD = 5.41 years) with methodology and sample size similar to others (Table 8). Given the results presented in this study, the question arises as to what is the cause of such a difference in results. A possible cause could be the software used for statistical analysis, given that most authors used the Statistical Package for Social Sciences for Windows, as in Kvaal’s study [10], unlike Bosman et al. [26], who used SAS univariate procedures (SAS statistical software—SAS Institute, Cary, NC, USA). Interestingly, Meinl et al. [27] reported results almost identical to the ones in the study of Bosman et al. using the same software [26,27] Owing to these mentioned facts, we processed the data with the SAS statistical software to check the possibility of software being the cause of more favorable results, but this proved to be an incorrect assumption, since we obtained identical results as when we processed the data with SPSS software (data not shown).

CBCT provides additional possibilities due to more significant measurement accuracy compared to the OPG recordings. The biggest impact on increasing measuring accuracy would be achieved using a three-dimensional visualization of teeth as well as the possibility of direct measurements on CBCT scans without the need to transfer those recordings to another program and measure them there [28].

In addition to the potential application of CBCT technology for these purposes, we should also consider applying a three-dimensional convolution network (3D CNN). This modern technology provides the ability to process images very quickly through software learning patterns and recognizes important image elements without human involvement [29].

## 5. Conclusions

Based on the obtained results, we can conclude that Kvaal’s method is not applicable to the age assessment of Serbian subjects, given that the error in predicting age is too large. On the other hand, modern radiographic technologies with high precision and accuracy such as cone beam computed tomography (CBCT) are increasingly used in dentistry, opening the possibility of applying the Kvaal method. With that in mind, regression analysis should be performed on measurements obtained by CBCT analysis, and these results could be compared to the results from OPG measurements in further studies.

## Figures and Tables

**Table 1:** Tooth measurements performed in this study.

Tooth Measurements Performed in This Study	
T	maximum tooth length (distance from the most occlusal aspect of the tooth to the root apex)
P	length of the pulp cavity (distance from the most occlusal aspect of the radiolucent pulp cavity to the root apex)
R	root length (distance from the mesial enamel–cementum junction to the root apex)
A	mesiodistal width of the pulp/root measured at the enamel–cementum junction level
B	mesiodistal width of the pulp/root measured at half the distance between enamel–cementum junction and half of the root length
C	mesiodistal width of pulp/root measured at half of the root length
M	mean of all measured values
W	mean value of tooth widths measured at points B and C
L	mean value of lengths measured at points P and R
W-L	difference in mean values obtained for parameters W and L

**Table 2:** Correlation coefficients between chronological age and the Kvaal dental ratios ^a^.

Ratio	Tooth (FDI)					
	11/21	12/22	15/25	32/42	33/43	34/44
T	−0.162	−0.126	0.059	−0.020	0.079	−0.042
P	−0.293 **	−0.240 **	0.035	−0.040	−0.002	−0.115
R	−0.069	−0.113	0.107	−0.008	0.062	−0.081
A	−0.060	0.028	0.016	−0.084	−0.163	−0.270 **
B	0.038	0.097	0.034	−0.006	−0.181 *	−0.139
C	0.050	0.037	0.070	−0.010	−0.190 *	−0.111
M	−0.167	−0.133	0.084	−0.047	−0.058	−0.160
W	0.046	0.069	0.054	−0.023	−0.189 *	−0.130
L	−0.198 *	−0.186 *	0.075	−0.025	0.029	−0.101
W-L	−0.206 *	−0.216 *	0.052	−0.017	0.108	−0.054

^a^ P, ratio between pulp and root length; T, ratio between tooth and root length; R, ratio between pulp and tooth length; A, ratio between pulp and root width at cement–enamel junction (level A); B, ratio between pulp and root width at midpoint between level A and level C (level B); C, ratio between pulp and root width at mid-root level (level C); M, mean value of all ratios; W, mean value of width ratios from levels B and C; L, mean value of P/R and P/T ratios. * *p* < 0.05; ** *p* < 0.01.

**Table 3:** Multiple regression models for estimation of chronological age (in years) from individual maxillary and mandibular teeth ^a^.

Tooth (FDI)	R	R^2^	Equation	SEE (±Years)
11/21	0.208	0.043	Age = 48.242 − 0.429(M) − 0.882(W-L)	12.443
12/22	0.226	0.051	Age = 42.867 + 1.207(M) − 1.698(W-L)	12.393
15/25	0.087	0.007	Age = 20.751 + 1.066(M) − 0.039(W-L)	12.676
32/42	0.058	0.003	Age = 39.148 − 1.202(M) + 0.280(W-L)	12.700
33/43	0.210	0.044	Age = 38.923 − 2.505(M) + 1.521(W-L)	12.437
34/44	0.178	0.032	Age = 54.865 − 3.017(M) + 0.704(W-L)	12.518

^a^ See Table 2.

**Table 4:** Comparative analysis of measured variables for all possible combinations ^a^.

Tooth (FDI)	Mean	SD	S.E.M.	*t*-Test
11/21	0.0075	12.9794	1.1428	ns
12/22	0.0032	12.9152	1.1371	ns
15/25	0.0084	12.5787	1.1075	ns
32/42	−0.0024	12.7406	1.1218	ns
33/43	−0.0124	13.1383	1.1568	ns
34/44	−0.0009	13.0175	1.1461	ns
3 Maxillary teeth (2 predictors)	−0.0017	13.1132	1.1546	ns
3 Maxillary teeth (6 predictors)	0.0067	13.3327	1.1739	ns
3 Mandibular teeth (2 predictors)	0.0022	12.9366	1.1390	ns
3 Mandibular teeth (6 predictors)	0.0015	13.1566	1.1584	ns
6 Teeth (2 predictors)	0.0075	12.9794	1.1428	ns
6 Teeth (12 predictors)	0.0120	13.5849	1.1961	ns

^a^ See Table 2 (mean, mean difference between chronological age and calculated age in years; S.D., standard deviation of this mean difference in years; S.E.M., standard error of the mean in years; * significant difference between calculated age and mean age; ns, no significant difference).

**Table 5:** Multiple regression models for the estimation of chronological age (in years) from the combined maxillary and mandibular teeth ^a^.

Tooth (FDI)	R	R^2^	Equation	SEE (±Years)
3 Maxillary teeth (2 predictors)	0.171	0.029	Age = 49.998 − 2.079(M) + 0.537(W-L)	12.534
3 Maxillary teeth (6 predictors)	0.315	0.099	Age = 36.303 − 2.198(11/21M) + 0.150(11/21W-L) + 1.326(12/22M) − 1.648(12/22W-L) +2.723(15/25M) + 0.045(15/25W-L)	12.269
3 Mandibular teeth (2 predictors)	0.213	0.045	Age = 42.606 − 1.114(M) + 0.300(W-L)	12.429
3 Mandibular teeth (6 predictors)	0.276	0.076	Age = 56.132 − 1.033(32/42M) − 0.329(32/42W-L) − 1.589(33/43M) + 1.985(33/43W-L) − 1.803(34/44M) − 0.076(34/44W-L)	12.428
6 Teeth (2 predictors)	0.208	0.043	Age = 48.242 − 0.429(M) − 0.882(W-L)	12.443
6 Teeth (12 predictors)	0.405	0.164	Age = 46.980 − 1.662(11/21M) − 0.108(11/21W-L) + 1.824(12/22M) − 1.553(12/22W-L) + 3.454(15/25M) − 0.197(15/25W-L) − 0.697(32/42M) − 0.083(32/42W-L) − 1.676(33/43M) + 1.787(33/43W-L) − 2.102(34/44M) − 0.134(34/44W-L)	12.120

^a^ See Table 2.

**Table 6:** Multiple backward regression models for the estimation of chronological age (in years) from the variables with the strongest correlation with age ^a^.

Variables	R	R^2^	Equation	SEE (±Years)
UWLAVG, 33/44B, gender, 33/44A, 12/22P, 11/21P, 33/43C, 11/21L, 12/22W-L, 12/22L	0.541	0.292	Age = 88.603 − 7.967(gender) − 2.109(11/21P) − 4.187(12/22P) − 4.394(34/44A) − 0.901(33/43B) − 0.034(33/43C) + 1.796(11/21L) + 5.750(12/22L) − 1.584(12/22W-L) − 0.407(UWLAVG)	11.057
UWLAVG, 33/44B, gender, 33/44A, 12/22P, 11/21P, 11/21L, 12/22W-L, 12/22L	0.541	0.292	Age = 89.639 − 7.968(gender) − 2.108(11/21P) − 4.188(12/22P) − 4.392(34/44A) − 0.935(33/43B) + 1.794(11/21L) + 5.754(12/22L) − 1.587(12/22W-L) − 0.406(UWLAVG)	11.011
33/44B, gender, 33/44A, 12/22P, 11/21P, 11/21L, 12/22W-L, 12/22L	0.540	0.292	Age = 90.444 − 7.936(gender) − 2.150(11/21P) − 4.197(12/22P) − 4.301(34/44A) − 0.914(33/43B) + 1.438(11/21L) + 5.989(12/22L) − 1.843(12/22W-L)	10.968
gender, 33/44A, 12/22P, 11/21P, 11/21L, 12/22W-L, 12/22L	0.537	0.289	Age = 88.464 − 7.795(gender) − 2.240(11/21P) − 4.039(12/22P) − 4.875(34/44A) + 1.577(11/21L) + 5.818(12/22L) − 1.919(12/22W-L)	10.947
gender, 33/44A, 12/22P, 11/21P, 11/21L, 12/22L	0.530	0.281	Age = 92.121 − 7.910(gender) − 2.482(11/21P) − 4.491(12/22P) − 4.191(34/44A) + 1.715(11/21L) + 4.562(12/22L)	10.962
gender, 33/44A, 12/22P, 11/21P, 12/22L	0.515	0.265	Age = 92.577 − 7.565(gender) − 1.219(11/21P) − 5.405(12/22P) − 4.106(34/44A) + 5.829(12/22L) ^b^	11.037

^a^ See Table 2; UWLAWG, maxillary average value of W and L difference; ^b^ for all coefficients *p* < 0.05.

**Table 7:** Tests of the accuracy of age estimation methods using a validation sample of digital OPGs with known age ^a^.

Tooth (FDI)	Mean Estimated Age (Years)	Min. Error of Estimate (Years)	Max. Error of Estimate (Years)	Standard Error of Estimate (±Years)
11/21	30.071	0.139	13.141	11.657
12/22	30.369	0.266	15.881	11.704
15/25	31.524	1.838	14.009	11.670
32/42	31.543	0.442	13.684	11.696
33/43	32.472	0.008	16.208	12.052
34/44	31.464	1.353	15.286	11.930
3 Maxillary teeth (2 predictors)	33.754	1.308	16.475	12.294
3 Maxillary teeth (6 predictors)	31.788	1.443	14.229	11.520
3 Mandibular teeth (2 predictors)	36.118	0.032	18.156	13.288
3 Mandibular teeth (6 predictors)	32.511	0.156	16.476	12.594
6 Teeth (2 predictors)	30.071	0.139	13.141	11.657
6 Teeth (12 predictors)	30.852	0.664	15.537	11.985

^a^ See Table 2 and Table 6.

**Table 8:** Authors who applied Kvaal’s method to different populations—comparison of the obtained standard deviations.

Study	Sample Size	Age Range (Years)	Population	Standard Deviation (Years)
Bosman et al. [26]	197	19–75	Belgian	5.41 (SD)
Meinl et al. [27]	44	13–24	Austrian	5.84 (SD)
Li et al. [13]	360	20–65	Chinese	11.8 (SD)
Kanchan-Talreja et al. [25]	100	25–77	Indian	14.7 (SD)
Erbudak et al. [23]	123	14–57	Turkish	11.82 (SD)
Roh et al. [24]	266	20–29	Korean	11.58 (SD)
Present study	170	17–77	Serbian	13.58 (SD)

## Data Availability

Data are available on request from the authors.
